# Spatio-temporal trends, distribution and prediction of tuberculosis incidence in Uganda (2020–2025)

**DOI:** 10.1186/s12879-025-12440-x

**Published:** 2025-12-25

**Authors:** Augustus Aturinde, Geofrey Amanya, Robinah Ikwangu

**Affiliations:** 1 Department of Geoinformatics, Centre for GIS and Spatial Computation, School of Built Environment, Kyambogo University , Kampala, Uganda; 2 National Leprosy and Tuberculosis Programme, Ministry of Health, Kampala, Uganda; 3 Infectious Disease Institute, Makerere University, Kampala, Uganda; 4 Department of Geomatics Engineering, Faculty of Engineering and Survey, Ndejje University, Luweero, Uganda

**Keywords:** Tuberculosis surveillance, Spatial epidemiology, Hotspot analysis, Spatiotemporal forecasting, Uganda

## Abstract

**Background:**

Tuberculosis (TB) remains a significant public health challenge in Uganda. As countries strive toward the End TB targets – a 90% reduction in TB deaths and an 80% reduction in TB incidence by 2030, timely data-driven insights are critical for guiding effective responses, particularly in the post-COVID-19 context.

**Methods:**

This study analysed district-level TB case notification data from January 2020 to February 2025, sourced from the National Tuberculosis and Leprosy Programme (NTLP). We applied temporal trend analysis, spatial autocorrelation, hotspot and emerging hotspot detection, and forest-based space-time forecasting to assess past patterns and project incidence through February 2026.

**Results:**

TB notifications increased sharply from 29,640 cases in 2020 to 72,768 in 2024, with case notification rates rising from 71.3 to 158.5 per 100,000 population. Spatial analysis revealed persistent clustering in northeastern Uganda (notably Moroto and Nakapiripirit), districts bordering Lake Victoria (e.g., Mukono, Buikwe, Buvuma), and emerging clusters in oil-rich Lake Albert regions. Cold spots were consistently identified in eastern and southwestern Uganda. Forecasts suggest a continued high burden in these same areas.

**Conclusion:**

By identifying stable, emerging and projected TB clusters, this study offers actionable insights to guide targeted interventions, optimize surveillance, and inform strategic planning. These findings provide a data-driven pathway toward accelerating Uganda’s progress toward its End TB commitments.

**Clinical trial:**

Not applicable.

## Introduction

Tuberculosis (TB) remains one of humanity’s oldest and most persistent infectious diseases, yet it is both preventable and curable [1, 2]. According to the Global Tuberculosis Report 2024, TB claimed over 1.25 million lives in 2023, and 10.8 million individuals developed the disease, making it once again the world’s deadliest infectious disease after briefly being eclipsed by COVID-19 [3].

Caused by *Mycobacterium tuberculosis* and primarily spread through respiratory droplets from individuals with active pulmonary TB, the disease continues to pose a serious global health burden. Approximately one-quarter of the world’s population is infected with latent TB [4], underscoring the pathogen’s threat to global health [5].

This disease burden and the severity of the epidemic, however, are not shared equally across the regions [6]. The Global Tuberculosis Report of 2024 shows that geographically, most people who developed TB were in the World Health Organisation (WHO) regions of South-East Asia (45%), Africa (24%), Western Pacific (17%), Eastern Mediterranean (8.6%), the Americas (3.2%) and Europe (2.1%) and that the 30 high TB burden countries, including Uganda, account for 87% of all estimated TB incidences [3].

Understanding these geographical disparities is critical to targeting effective interventions. In Uganda, TB continues to impose significant public health and economic costs [7], despite the rollout of advanced diagnostic tools such as Xpert MTB/RIF Ultra and expanded treatment programmes. In 2023, the country reported an estimated TB incidence of 200 cases per 100,000 population [8], with wide regional disparities driven by poverty, high HIV prevalence, urban crowding, and inadequate access to healthcare [9].

The Uganda National Strategic Plan for Tuberculosis and Leprosy Control (2020–2025) highlights the role of spatial data in improving case detection through the use of GIS mapping to identify and target screening in TB hotspots areas [9]. Spatial analyses, particularly those integrating epidemiological and geospatial data, are therefore critical for identifying hotspots, understanding TB drivers, and guiding targeted interventions [10].

While traditional spatial analysis has improved understanding of TB distributions, its typically static nature limits the assessment of how disease patterns evolve. Space-time analytical methods address this limitation by capturing the dynamic interaction between location and time, thereby enhancing predictions of when and where TB is likely to emerge [11, 12]. Recent applications in tracking diseases such as COVID-19 have demonstrated the power of these methods to inform timely public health responses [13, 14].

Despite growing international interest in space-time analytics, their application to TB distribution research in Uganda remains limited. This study addresses this gap by providing a comprehensive spatial and temporal profile of TB notifications in Uganda from 2020 to 2025, including forecasts through February 2026. In doing so, it contributes critical insights that can guide evidence-based policy, optimise resource allocation, and accelerate Uganda’s progress toward the End TB goals as reaffirmed at the 2023 United Nations High-Level Meeting.

This study has two main objectives:

1. To profile the spatial and temporal distribution of TB notification in Uganda from 2020 to 2025, during and after the COVID-19 pandemic; and.
2. To forecast TB incidence and hotspot clustering across districts between March and February 2026.

## Methods

### Data sources

Population data was downloaded from the Uganda Bureau of Statistics. While population projections were used for the non-census year of 2020—2023, the official district populations were used for 2024.

Weekly TB notification data (new and relapse cases diagnosed and registered), aggregated into monthly records, was obtained from the Ministry of Health National Leprosy and Tuberculosis Programme (NLTP) database for the 62 months of analysis (January 2020 to February 2025).

Administrative boundary data was also obtained from the Uganda Bureau of Statistics. Given that most city boundaries are not mapped, the statistics for all new cities were merged back to their original districts (save for Kampala).

### Scale of analysis

The monthly TB case notification records for the 62 months of analysis were summarized across Uganda as a country, regionalised into the four regions (central, west, east and north) and later locally analysed at the district level. The smallest spatial unit of analysis was the district level while the finest temporal unit was a month.

### Statistical and Spatial analysis

#### Computation of case notification rates (CNRs)

District-specific monthly TB case notifications were aggregated to obtain annual counts for the period 2020–2024. Using corresponding district population estimates from Uganda Bureau of Statistics, case notification rates (CNRs) per 100,000 population were calculated as:

1 $$CN{R_{i,y}} = \left( {\frac{{\mathop \sum \nolimits_{m = 1}^{12} T{B_{i,m}}}}{{Po{p_{i,y}}}}} \right) \times 100,000$$

where:

$\:{CNR}_{i,y}$ is the case notification rate for district $\:i$ in year $\:y$,

$\:{TB}_{i,m}$ is the number of TB cases reported in district $\:i\:$in month $\:m$,

$\:{Pop}_{i,y}$ is the district population in year $\:y$.

These CNRs were summarized at national and regional levels and visualized using line and bar charts to illustrate trends and regional variation in TB burden.

#### Choropleth mapping

Annual district-level CNRs were visualized using choropleth maps constructed with the Natural Breaks (Jenks) classification method. This approach facilitated the identification of spatial heterogeneity and potential spatial clusters in TB distribution across districts for each year.

#### Global moran’s index

To assess the overall spatial autocorrelation in TB case notification rates across Uganda, Global Moran’s Index (I) was computed annually for the period 2020–2024 using the following formula:

2 $$I = \frac{n}{W} \times \frac{{\mathop \sum \nolimits_{i = 1}^n \mathop \sum \nolimits_{j = 1}^n {w_{ij}}\left( {{x_i} - \bar x} \right)\left( {{x_j} - \bar x} \right)}}{{\mathop \sum \nolimits_{i = 1}^n {{\left( {{x_i} - \bar x} \right)}^2}}}$$

where:

$\:n$ is total number of spatial units (districts)

$\:{x}_{i}$ is the value of CNR at district$\:\:i$

$\:\stackrel{-}{x}$ is the mean of CNR

$\:{w}_{ij}$ is the spatial weight between district $\:i\:$and $\:j$

$\:W=\sum\:_{i=1}^{n}\sum\:_{j=1}^{n}{w}_{ij}$ is the sum of all spatial weights

Under spatial randomness, the expected Moran’s I is given by the formula:

3 $$E\left[ I \right] = \frac{{ - 1}}{{n - 1}}$$

Variance of Moran’s I is given by the formula:

4 $$Var\left[ I \right] = \frac{{{S_1} - E{{\left[ I \right]}^2}}}{{\left( {n - 1} \right)\left( {n - 2} \right)}}$$

where $\:{S}_{1}$ is the square of the spatial weights.

The Z-score is given by the formula:

5 $$z = \frac{{I - E\left[ I \right]}}{{\sqrt {Var\left[ I \right]} }}$$

#### Hotspot analysis: Getis-Ord Gi*

To detect statistically significant spatial clusters of high or low TB CNRs, Getis-Ord Gi* statistics were computed at the district level. This local spatial autocorrelation method identifies “hotspots” (high-value clusters) and “cold spots” (low-value clusters) using the following formula:

6 $$G_i^* = \frac{{\mathop \sum \nolimits_{j = 1}^n {w_{i,j}}{x_j} - \bar X\mathop \sum \nolimits_{j = 1}^n {w_{i,j}}}}{{S\sqrt {\frac{{n\mathop \sum \nolimits_{j = 1}^n w_{i,j}^2 - {{\left( {\mathop \sum \nolimits_{j = 1}^n {w_{i,j}}} \right)}^2}}}{{n - 1}}} }}$$

where:

$\:{x}_{j}$ is the CNR in district j,

$\:{w}_{i,j}$ is the spatial weight between district $\:i\:$and $\:j$,

$\:n$ is the total number of districts,

$\:\stackrel{-}{X}$ is the mean CNR, and.

$\:S$ is the standard deviation of the CNRs.

#### Space-time emerging hotspot analysis

A space-time cube was constructed using district-level CNR data, with each cube “bin” representing a single district-month combination (January 2020–February 2025). The cube captured the evolution of TB burden both spatially and temporally using the space-time implementation of the Getis-Ord Gi* statistic.

**Fig. 1:**
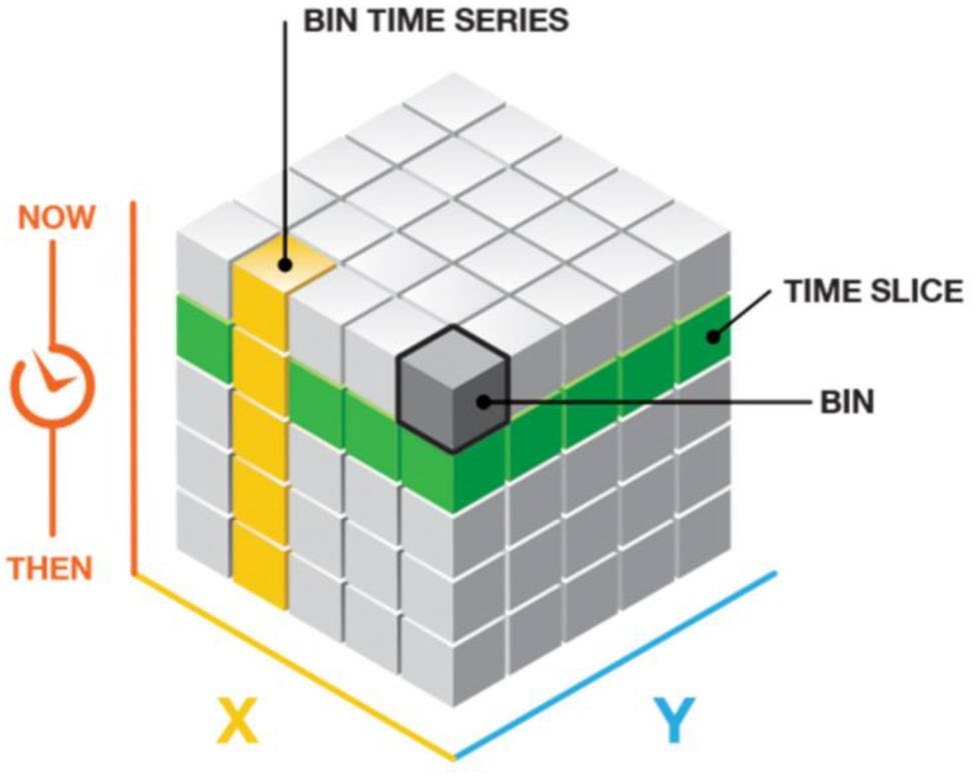
space-time cube (from https://pro.arcgis.com/)

Each bin was assigned a location ID (district), a time step (month), and a variable value (CNR). Bins were grouped: (1) spatially into districts, (2) temporally into time slices, and (3) longitudinally into time series for trend detection (see Fig. 1).

Trend analysis of the resulting z-scores was performed using the Mann-Kendall trend test, a non-parametric rank correlation statistic. It assessed the presence of monotonic trends in TB burden over time. Z-scores and p-values from this test determined the classification of each district’s TB pattern into one of 18 categories, such as new, intensifying, persistent, sporadic, or diminishing hot/cold spots.

#### Space-time forecasting using forest-based regression

To forecast TB CNR for the period March to February 2026, this study applied forest-based space-time forecasting, a machine learning method that uses an ensemble of decision trees (random forest) to model non-linear temporal trends at the district level. The method fits a regression model where the dependent variable is the monthly CNR and the predictor is the time step (i.e., the month). Using this model, future values are generated recursively – each predicted value informs the subsequent forecast.

The model was trained using 62 months of historical district-level CNR data (January 2020 to February 2025). To evaluate model performance, the final six months of this series were withheld and used for out-of-sample validation. Forecast accuracy was assessed using the Root Mean Square Error (RMSE), which quantifies the average deviation between predicted and observed values.

The model accommodates non-linear trends and irregular temporal patterns, making it suitable for forecasting public health data with complex seasonal and spatial dependencies. A comprehensive description of the algorithm and its implementation can be found elsewhere [15].

## Results

### Trends within the TB notification data (2020–2025)

Overall, tuberculosis notifications in Uganda showed a consistent upward trend between 2020 and 2025. The total number of reported cases increased from 29,640 in 2020 to 43,105 in 2021, 59,526 in 2022, 61,731 in 2023, and 72,768 in 2024. In the first two months of 2025 alone, 9,374 cases were recorded. Across the 62-month analysis period (January 2020 to February 2025), a cumulative total of 276,144 TB case notifications was reported.

**Fig. 2:**
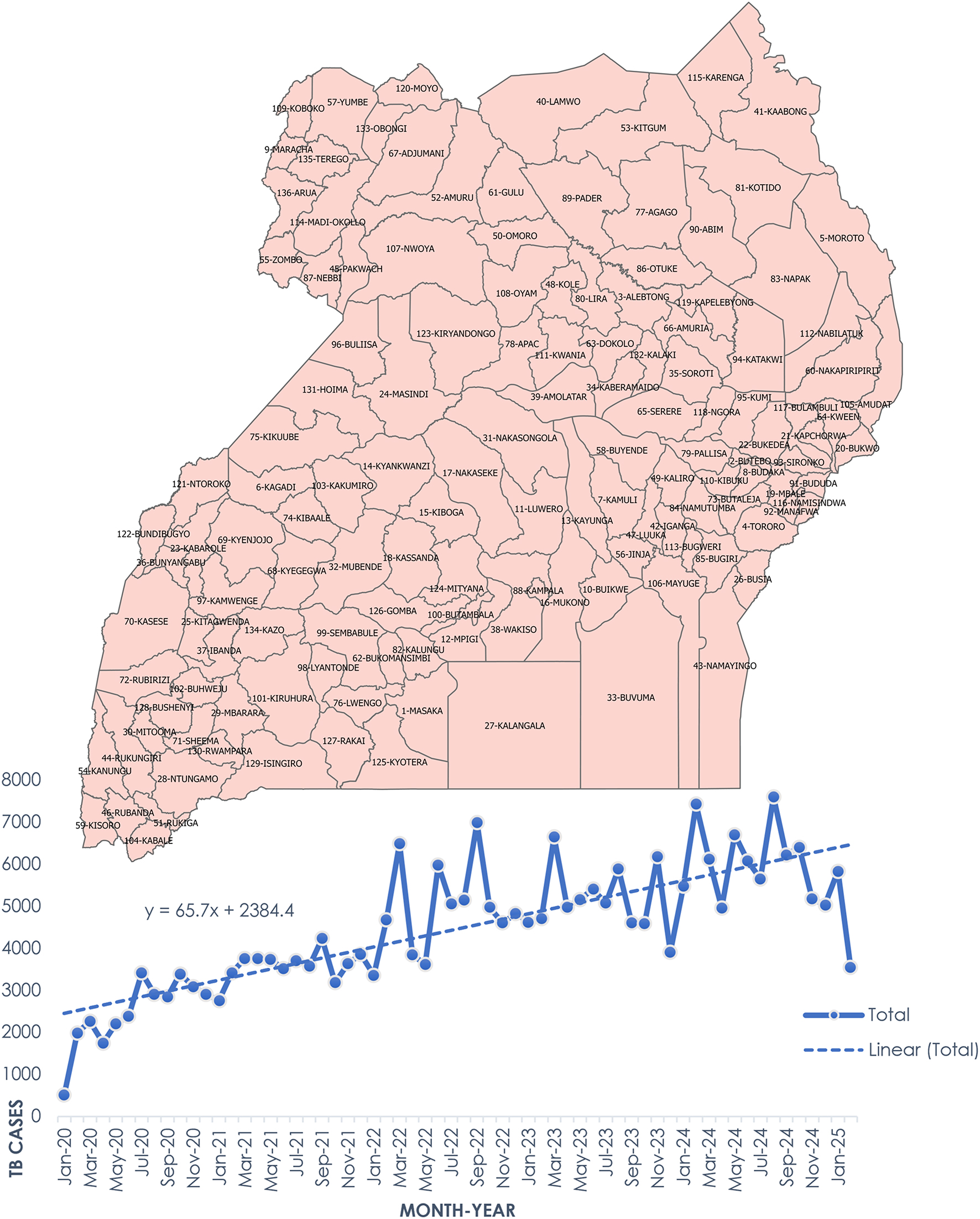
Monthly tuberculosis (TB) notification trends by district in Uganda (January 2020–February 2025). Districts are labelled with their unique identification numbers preceding the district names (e.g., 5-Moroto)

**Fig. 3:**
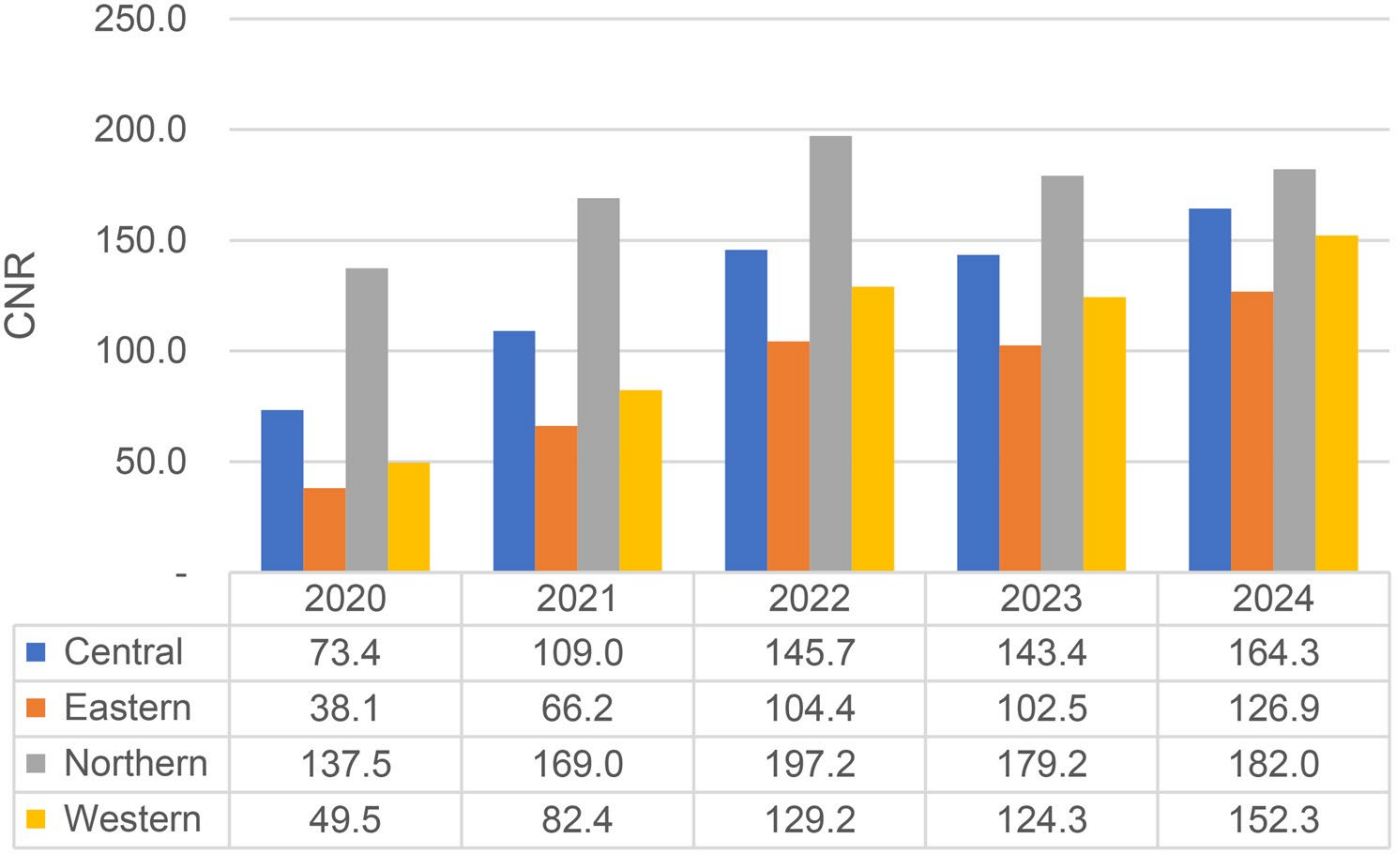
CNR across the 4 regions of Uganda (2020–2024)

The calculated CNR per 100,000 population similarly exhibited an upward trend as shown by Fig. 2. Regionalised CNR per 100,000 population show that consistently the Northern region has the highest, followed by Central, Western and finally Eastern with the lowest as shown in Fig. 3.

### Spatial distribution of TB incidence

**Fig. 4:**
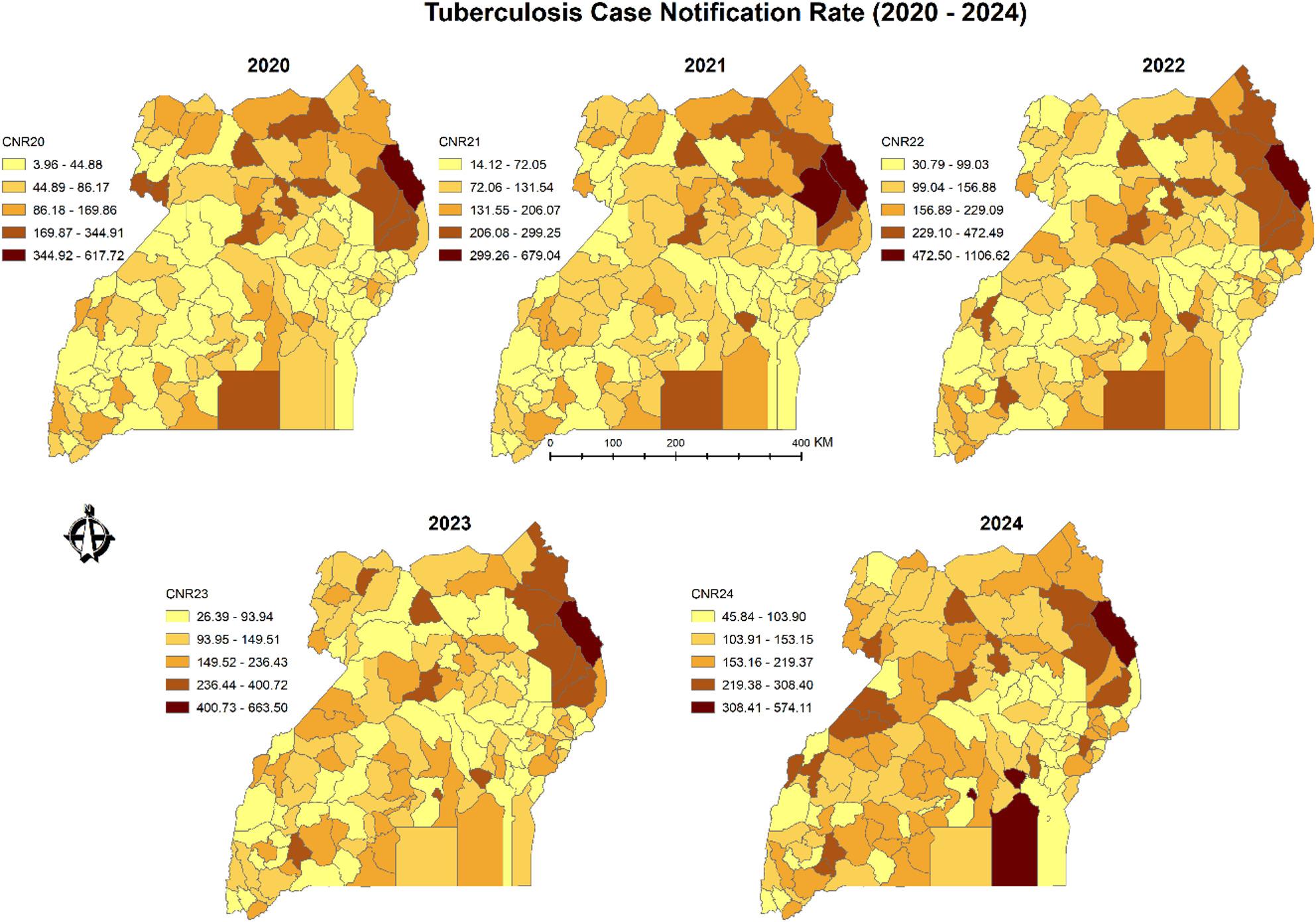
Annual tuberculosis (TB) case notification rates in Uganda, 2020—2024, classified into five categories using the Natural Breaks (Jenks) method

To further examine regional disparities in TB incidence, choropleth maps were generated to visualize district-level CNR across Uganda for each of the four years under analysis. As shown in Fig. 4, higher CNRs were consistently observed in the greater northern region, particularly in the northeastern districts, and in the southern lake districts of Buvuma and Kalangala. Elevated rates were also evident in districts hosting major urban centres, including Kampala, Jinja, Mbale, Mbarara, Gulu, and Hoima.

### Global Moran’s index

Spatial autocorrelation analysis using Global Moran’s Index revealed a generally positive and significant clustering of TB case notification rates across districts from 2020 to 2023 (Table 1), with Moran’s I values ranging from 0.7164 (2020) to 0.0936 (2023). Although the index for 2024 remained positive (I = 0.0780), it was not statistically significant (*p* = 0.0603), suggesting a weakening in spatial clustering that year.

**Table 1:** Moran’s indices for the yearly TB case notification rates (2020–2024)

Year	Moran’s I	z-score	*p*-value	Variance
2020	0.7164	4.2470	0.0000	0.0018
2021	0.1774	4.2228	0.0000	0.0019
2022	0.1065	2.9578	0.0031	0.0015
2023	0.0936	2.2601	0.0238	0.0020
2024	0.0780	1.8784	0.0603	0.0021

### Spatial clustering of TB incidence

**Fig. 5:**
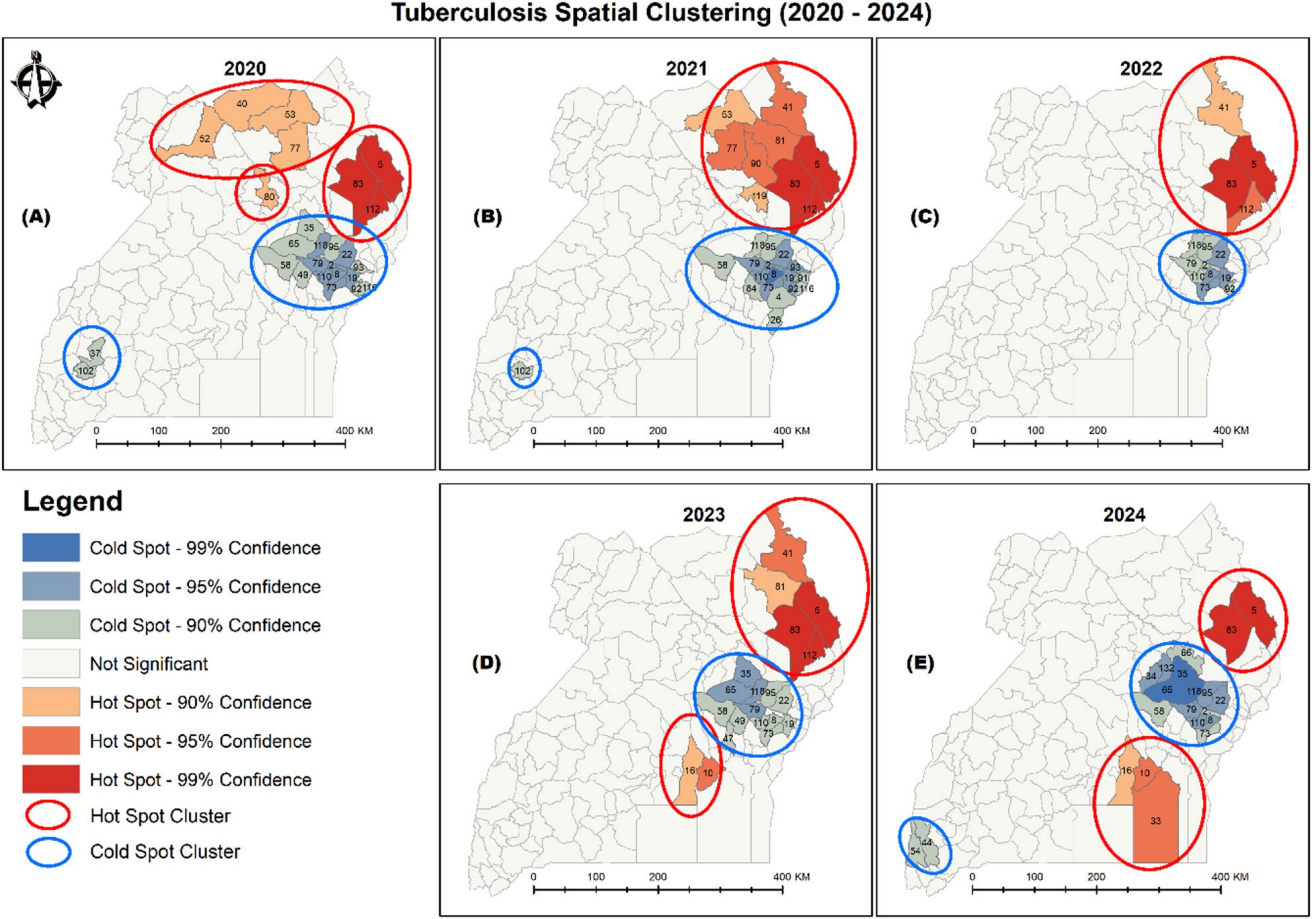
Tuberculosis spatial clusters (2020–2024). District unique identity numbers indicated per cluster

To better understand the spatial heterogeneity of TB burden, hotspot analysis was conducted to identify statistically significant clusters of high and low case notification rates across districts as shown in Fig. 5.

Figure 5 shows that in 2020, three closely located hotspot clusters emerged in the greater Northern region, encompassing eight districts. Conversely, cold spots were identified in the Eastern and Western regions, spanning 18 districts in total. In 2021, a single hotspot cluster appeared in the northeast, covering nine districts, while two cold spot clusters were identified – 17 districts in the East and one district in the West. By 2022, the hotspot cluster had contracted to four northeastern districts, while the cold spot cluster persisted in the East, covering 10 districts. In 2023, two distinct hotspot clusters emerged in the northeastern and central regions, involving seven districts in total, with a corresponding cold spot cluster in 13 eastern districts. In 2024, two hotspot clusters were again identified – one in the northeast and another in the southeastern part of the Central region, together comprising five districts. Additionally, cold spot clusters were detected in both the Eastern and southwestern Western regions, involving 16 districts.

### Emerging and forecasted TB incidence

While the hotspot and cold spot clusters shown in Fig. 5 represent retrospective spatial patterns, a space-time cube analysis was used to identify emerging trends in TB incidence across Uganda. This analysis, based on netCDF-format space-time bins, evaluates both spatial and temporal clustering using the Getis-Ord Gi* and Mann-Kendall statistics.

**Fig. 6:**
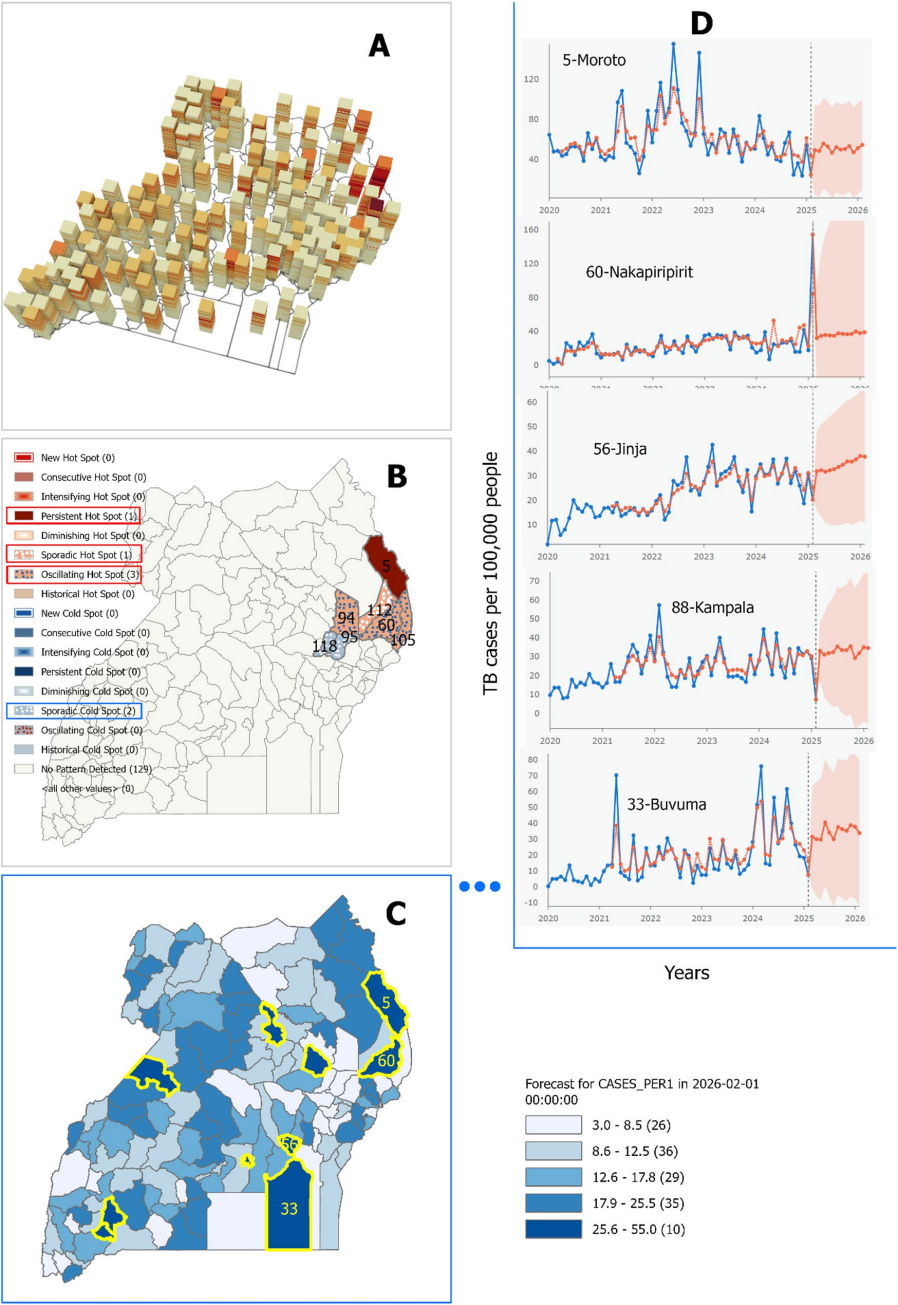
Space-time analysis and forecast of tuberculosis (TB) case notification rates (CNR) in Uganda. Panel **A**: Space-time cube of monthly CNR (2020–2025); Panel **B**: Emerging hotspot analysis; Panel **C**: Forecasted CNR (March 2025–February 2026) using forest-based modelling; Panel **D**: Forecast time series for the five districts with the highest projected CNR in February 2026. Brackets in the legend show the number of districts in each Natural Breaks (Jenks) classification. Districts are identified by unique numbers in Panels B, C, and D

Panel A of Fig. 6 visualizes the space-time cube for all 136 districts. Although three-dimensional structures are challenging to interpret visually, districts in the northeast (e.g., Moroto, Napak) exhibit darker bin colours near the top of the cube, indicating elevated TB CNRs in recent months.

Panel B presents the results of the emerging hotspot analysis (k-nearest neighbour = 4). Most districts (129/136) exhibited no statistically significant spatiotemporal pattern. However, seven districts showed meaningful clustering: (1) Persistent hotspot (Moroto); (2) Sporadic hotspot (Nabilatuk); (3) Oscillating hotspots (Katakwi, Nakapiripirit, Amudat); (4) Sporadic cold spots (Ngora, Kumi). These findings point to a geographically concentrated emerging TB burden in the northeast subregion.

Panel C displays the forecasted monthly TB CNRs to February 2026 using the forest-based space-time model. Model performance evaluation via the root mean square error (RMSE) showed no significant spatial autocorrelation in prediction error (Moran’s I = 0.0019; z = 0.227; *p* = 0.82), indicating a spatially uniform forecast error distribution.

Pane D highlights the 10 districts with the highest projected CNRs in February 2026, with the top five – Moroto, Nakapiripirit, Kampala, Jinja, and Buvuma labelled. Time series patterns show that Moroto, Jinja, Kampala, and Buvuma have had persistently elevated CNRs over the last five years. Notably, Nakapiripirit, historically low in CNR, shows a sharp recent uptick (in December 2024), suggesting a need for further epidemiological scrutiny.

### Forecasted spatial clusters of TB incidence (March-February 2026)

**Fig. 7:**
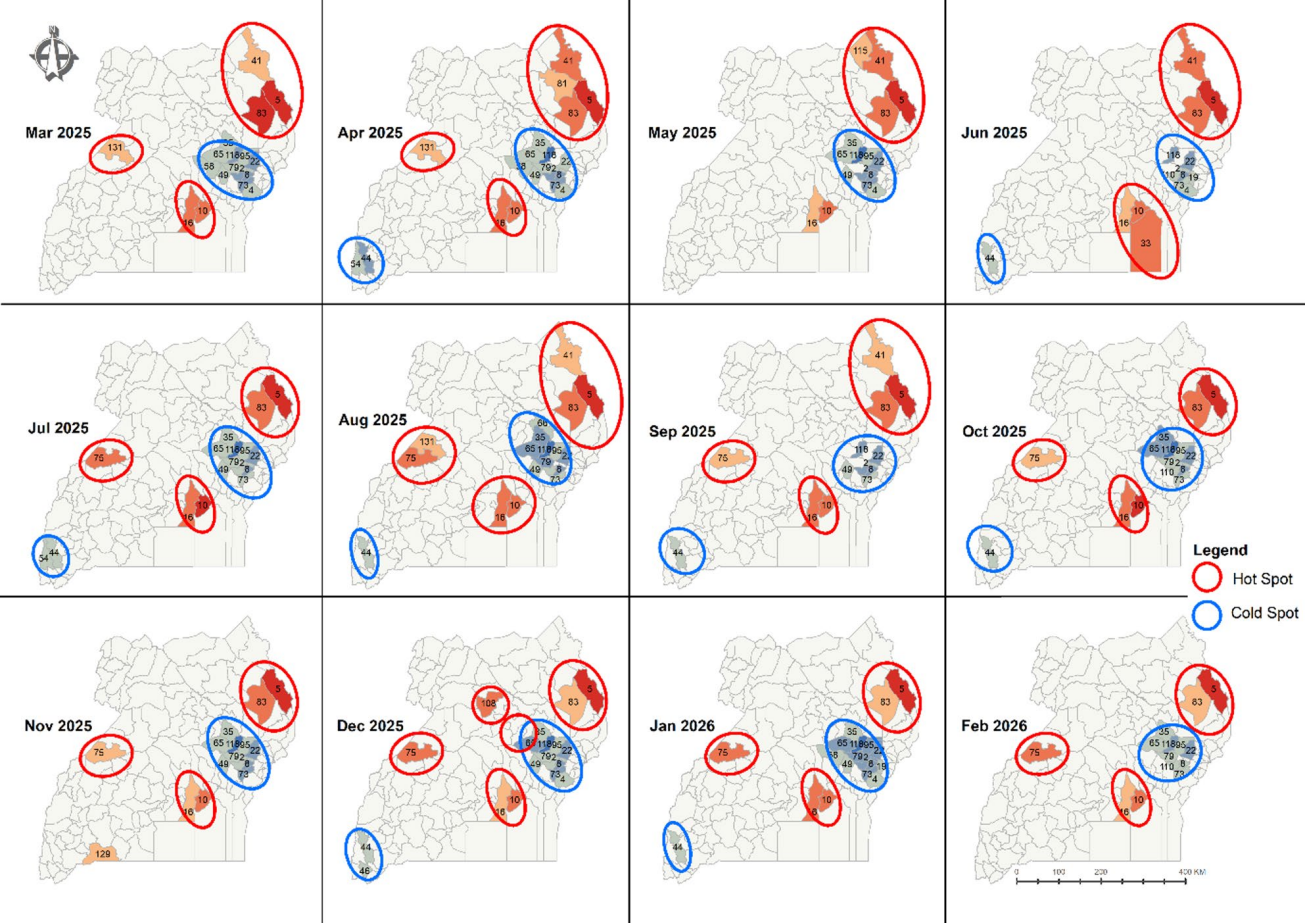
Forecasted TB CNR Hot spots and Cold spots (March 2025 to February 2026)

Building on the space-time forecast, Fig. 7 presents the hotspot and cold spot analysis of forecasted TB case notification rates across Uganda for the period March to February 2026.

Figure 7 identifies three persistent hotspot regions: (1) the Northeastern subregion, (2) the Central-Western corridor, and (3) the Southeastern coastal districts near Lake Victoria. Although the spatial extent of these clusters fluctuated slightly from month to month, their geographical cores remained stable throughout the twelve-month forecast period.

Cold spot clusters were predominantly *located in the Eastern region*, with an additional small cold spot emerging in the Southwestern highlands. Similar to the hotspots, the cold spot areas showed minor changes in size but remained geographically consistent over time.

Overall, the forecast suggests spatial stability in both high and low TB incidence zones, reinforcing the patterns identified in retrospective and emerging hotspot analyses. This stability highlights critical regions for targeted TB interventions over the next operational cycle.

## Discussion

The End TB Strategy aims to reduce TB deaths by 90% and TB incidence by 80% by 2030, relative to 2015 levels. Yet, by 2023, the global reduction in TB incidence stood at just 8.3%, far below the interim target of 50% by 2025 [3]. Achieving these ambitious goals demands not only intensified efforts but also targeting interventions where they are most needed and most likely to yield. Spatial and spatiotemporal analyses have proven essential in this regard, uncovering geographic disparities in disease burden and illuminating local drivers of transmission that might otherwise remain hidden [16].

Our findings (Figs. 2 and 3) indicate an increase in TB notification counts and case notification rates from 2020 to 2025. This trend mirrors that of the WHO African Region, where TB notifications rose during and after the COVID-19 pandemic, contrary to global declines [3]. The relative stability of TB detection in Africa has been attributed to minimal disruption from COVID-19-related health system shocks, though the possibility of over-diagnosis in some settings cannot be ruled out [3]. Similar rise in TB CNR between 2013 and 2022 have previously been reported [8], reinforcing the pattern observed in this study. Notably, a brief stagnation between 2022 and 2023 may reflect delayed effects of the pandemic; however, the sharp increase in 2024 suggests a recovery in TB case detection. This resurgence is likely linked to the nationwide community-based active case-finding campaign (CAST TB), launched in 2022, which focuses on awareness, screening, testing, prevention, and treatment at the community level [17]. It may also attributed to a general global recovery in access to diagnosis and treatment post-pandemic [3].

Choropleth mapping of TB case notification rates (Fig. 4) reveals clear spatial heterogeneity across Uganda, with distinct areas of high, medium, and low burden. Regions with disproportionately high TB CNRs are concentrated in the northeast, greater north, far west, and south eastern regions – patterns that align with findings from prior research [8, 18, 19]. This uneven distribution reflects a complex mix of factors, including disparities in health-seeking behaviour shaped by capacity, opportunity, and motivation [20]; limited health system resources; gaps in data quality; health worker morale; and widespread poverty [8]. Together, these factors help explain why certain regions remain entrenched in a cycle of high TB burden.

The spatial distribution of TB case notification rates exhibited significant clustering, as confirmed by Global Moran’s Index (Table 1). Clustering was strongest in 2020 (I = 0.7164, *p* < 0.001), but declined progressively through 2023 (I = 0.0936, *p* = 0.024). By 2024, the index remained positive (I = 0.0780) but was no longer statistically significant (*p* = 0.060), indicating a possible spatial diffusion of TB or weakening of previously persistent clusters. This attenuation may reflect post-COVID changes in transmission patterns [21] or expanded case detection in previously low-incidence areas [22, 23]. Increasing (early) TB detection allows for prompt interventions, reduces risk of severe complications, and curbs the transmission chain by enabling identification and isolation of infected persons [24].

Figure 5 reveals persistent hot and cold spot spatial clusters of tuberculosis that align closely with historical patterns and previous studies on TB spatial dynamics [8, 19], suggesting a tendency for high- and low-burden areas to persist over time. The Karamoja subregion is critical [25] with a TB notification rate of approximately 450 per 100,000 – more than double the national average of 213 per 100,000 within a population of about 1.5 million [26]. This regional high burden is driven by a combination of food insecurity/undernutrition, overcrowding, and poor housing ventilation [27], which may be worsened by the effects of climate change [28]. The 2024 national census highlights Karamoja as the region with the lowest human development indicators in Uganda [26]. Dominated by pastoralist communities, Karamoja’s communal and densely clustered settlements, along with seasonal migration patterns, significantly increase TB transmission and contribute to treatment default, thereby exacerbating the disease’s persistence and spread [25].

Figure 5 also reveals persistent TB hotspot clusters in the southeastern districts of Buikwe, Mukono, and Buvuma areas in/adjacent to Lake Victoria and predominantly inhabited by fishing communities. These populations face heightened vulnerability due to structural barriers such as limited access to healthcare, poor treatment adherence, and high mobility, which collectively hinder early TB diagnosis and treatment [29]. Critically, these same communities also bear a disproportionate burden of HIV, a well-established driver of TB incidence in Uganda [9]. The overlap between high HIV prevalence and TB notification rates in these districts suggests a strong syndetic interaction, where TB-HIV coinfection likely sustains and amplifies the observed clusters [19]. As Bolton [30] asserts, “the highest numbers of this co-infection are found around Lake Victoria and in northern Uganda.” These findings emphasize the urgent need for spatially targeted and integrated TB-HIV interventions, particularly within mobile, underserved populations such as fishing communities.

The presence of cold spot clusters may reflect one of two underlying realities: either a genuinely low TB incidence within these populations, or inefficiencies in case detection and reporting, where active TB cases remain undiagnosed or unnotified [31]. Given the relatively comprehensive coverage of the NTLP in Uganda’s District Health Information Surveillance system, it is more plausible that these cold spots correspond to districts with truly low TB burden [9]. Nonetheless, this interpretation should be approached with caution and further investigation into diagnostic access, health-seeking behaviour, and facility-level reporting practices in cold spot regions would help distinguish between low incidence and under-detection.

The space-time analysis in Fig. 6 (panel B) reveals four distinct categories of emerging TB hotspots within the same northeastern region. Notably, Moroto was classified as a persistent hotspot, consistent with earlier studies and national reports [8, 9]. While Nabilatuk, previously identified as a hotspot, was reclassified as a sporadic hotspot, the districts of Katakwi, Nakapiripirit, and Amudat emerged as oscillating hotspots. This temporal fluctuation may reflect the mobile, agropastoral lifestyles of residents in these areas, which influence both exposure risk and healthcare-seeking behaviour [25]. In contrast, the eastern districts of Ngora and Kumi were identified as sporadic cold spots, aligning with the broader pattern of cold spot clustering in that region. Importantly, the classification of hotspots and cold spots into subtypes adds analytical depth to spatial epidemiologic studies, enabling more precise targeting of interventions based on temporal stability and risk volatility [32].

The forecast of TB notification rates through February 2026 identified districts projected to carry the heaviest burden namely Moroto and Nakapiripirit in the Northeast, Jinja in the East, and Kampala and Buvuma in Central Uganda (Fig. 6 panel C and D). These projections align with patterns previously reported in national surveillance data and recent studies [9, 18, 33]. The model demonstrated strong predictive performance, with a low average root mean square error (RMSE) of 6.7 and no evidence of spatial autocorrelation in the residuals, indicating that the predictions are statistically sound and geographically unbiased [15]. These insights offer opportunities for strategic intervention highlighting exactly where resources and efforts are likely to yield the greatest impact as Uganda accelerates toward its End TB goals.

Beyond surface-level interpretations of predicted TB case notification rates, we conducted hotspot analyses on projected values for the period March to February 2026 (Fig. 7). This allowed us to pinpoint priority areas for intervention. Unsurprisingly, consistent with earlier findings of what is reported in the annual NTLP reports, and prior studies, the most critical regions include northeastern districts around Moroto, the islands and districts north of Lake Victoria such as Buvuma, and those surrounding Lake Albert.

Just as important, however, are the persistent cold spots in eastern Uganda and isolated parts of the southwest. These may indicate either the effectiveness of existing TB control interventions or reflect under-detection and reporting inefficiencies in the TB surveillance system. As Uganda intensifies its efforts to meet the End TB targets, the strategic value of these insights lies in their ability to guide timely, place-based interventions that ensure no region is left behind. This study therefore offers both a diagnostic mirror and a forward-looking map, one that can help policymakers and health authorities sharpen their focus where the burden is greatest and vigilance where the silence might be misleading. Importantly, forecast-driven decisions, especially in resource planning and allocation, should be made in conjunction with community level assessments to avoid unintended allocation bias [34].

### Limitations

While this study successfully profiled TB notification dynamics across Uganda and forecasted trends through February 2026, it did not account for potential explanatory factors behind the observed spatial and temporal patterns. Incorporating such variables, particularly at localized levels, could enhance understanding of the underlying drivers of TB incidence. Additionally, the study relies on reported TB data from the NTLP; any inconsistencies or underreporting within these records inevitably influence our results. Finally, as with all ecological studies, there is a risk of ecological fallacy – the danger of drawing conclusions about individuals based on aggregated data.

### Recommendations

The insights generated by this study are valuable to Uganda’s Ministry of Health and other stakeholders involved in TB control and resource planning. By identifying high-burden areas and forecasting near-future trends, the study offers timely guidance for targeted epidemiologic response and strategic planning. Public health officials should focus diagnostic and treatment efforts in predicted hotspots, particularly the northeast, southeast, and Lake Albert regions while also investigating persistent cold spots for possible surveillance gaps. The research community is encouraged to build upon these findings, especially by integrating explanatory variables such as HIV prevalence, poverty and deprivation indices, occupation and livelihood, and housing quality, to enhance model accuracy and public health relevance. Further, future work could also explore cross-border mobility, climate variability, and socio-behavioural dimensions affecting TB dynamics.

## Conclusion

This study provides the first comprehensive post-COVID-19 analysis of TB notification trends in Uganda, combining statistical, spatial, and predictive modelling to illuminate evolving disease dynamics from 2020 to 2025. TB case notifications have steadily increased, with marked spatial heterogeneity revealing persistent hotspots in the northeast, southeast, and around Lake Albert. These patterns highlight the need for region-specific interventions that address both current and forecasted burdens. However, despite the strong surveillance system, it is important to note that some observed and predicted cold spots, particularly in the eastern region, may be driven by under-detection rather than purely low incidence [35].

Encouragingly, the predictive model validated with high accuracy – pinpoints where timely, evidence-based action could accelerate progress toward the End TB 2030 targets. According to WHO, universal diagnosis and treatment within a single year could reduce TB incidence by up to 85%. This underscores the urgent need to scale up contact tracing, case notification, and treatment initiation. By identifying the “where” and the “when” of Uganda’s TB burden, this study lays a critical foundation for responsive, equitable, and impactful TB control.

## Data Availability

Data collected from the NTLP can be shared upon reasonable request from the corresponding author. Administrative and population data from the Uganda Bureau of Statistics are publicly available.

## Abbreviations

CAST: Community-based Active Case-finding Campaign
CNR: Case Notification Rates
DHIS2: District Health Information Surveillance system
HIV: Human Immunodeficiency Virus
MoH: Ministry of Health
NTLP: National Tuberculosis and Leprosy Programme
RMSE: Root Mean Square Error
TB: Tuberculosis
WHO: World Health Organisation
